# Transactions of the New York State Medical Society

**Published:** 1845-09

**Authors:** 


### BIBLIOGRAPHICAL NOTICES.

*Transactions of the New York State Medical Society.*—We have been favored, by Dr. T. R. Beck, of Albany, with a copy of Vol. VI, parts 1 and 2, of the Transactions of the New York State Medical Society. This volume contains the Transactions of the Society for the last two years, besides numerous interesting addresses and papers upon various medical subjects.

At the meeting of the Society in February last, the following Circular and Resolutions received much attention:

*To Physicians, Medical Colleges, and Medical Societies throughout the State.*—At the last annual meeting of the N. Y. State Med. Soc., a resolution was passed directing the standing corresponding committee to enquire into the expediency of separating entirely the business of *Teaching* and *Licensing*, in the medical profession—of lessening the number of boards for examining and licensing candidates, by inducing the several county medical societies to surrender their right to appoint censors for that purpose—and of simplifying the whole law regulating the practice of medicine and surgery in this State. The committee was also directed to prepare, at an early day, a Circular addressed to the several county medical societies, containing the changes which they intended to recommend, that the same might be duly considered and acted upon by those societies previous to the next annual meeting of the State Society. In accordance with the above directions, the committee have prepared, and would recommend to the mature consideration of the whole profession in this state, the following resolutions. They would recommend that the laws incorporating the state and county medical societies remain untouched, except the slight modifications required in the annexed resolutions; but in the place of all other laws regulating the practice of medicine, with their pains and penalties, they would substitute a few simple provisions, embodying the objects set forth in the resolutions already referred to. The chairman of the committee would respectfully request all who may receive this Circular to aid in bringing the subject before the medical societies and colleges in this state. And any communications which the members of the committee, and others, will make on this subject, will be gladly received by the chairmain.

*Resolved*, That the cause of medical education requires that the present numerous boards for examining and licensing candidates to practice medicine and surgery, composed of county and state censors, and the professors in our medical colleges, should be abandoned, and their place supplied by a single board of censors in each senatorial district in the State, to be appointed by the State Society, whose duty it shall be to meet annually, and remain in session one week, for the purpose of examining all candidates for license to practice physic and surgery in their respective districts; and the said censors to be paid a reasonable compensation out of the funds of said Society.

*Resolved*, That instead of requiring each candidate to study a required number of years, or in a prescribed place, he should be required to give satisfactory evidence to the board before which he is examined, that he possesses a *thorough* knowledge of every branch of medical science; that he has personally assisted in the dissection of at least two good anatomical subjects, under the direction of a regular practising physician, or in some incorporated medical college in this state; that he is practically acquainted with chemistry and botany, and has presented the board with an essay on some medical subject, written by himself; that he is twenty-one years of age, and of good moral character.

*Resolved*, That a second and honorary degree be instituted in the medical profession, to be conferred by the Regents of the University of the State of New York, on the recommendation of a district board of censors. But no candidate shall be admitted to an examination for the honorary degree, until he shall have practised medicine and surgery three years, given evidence of a thorough knowledge of general and comparative anatomy, and presented the board of examination with an essay on some medical subject, written in the English, French and German languages.

N. S. DAVIS,
*Chairman of Committee.*

These resolutions were reported upon favorably by a majority of the committee of Correspondence. Dr. M. H. Cash, from the minority of the same committee, reported adverse. The two reports ably discuss the subject pro and con. The opinions of the county societies with regard to these resolutions may be gathered from the following:

The committee to whom was referred the communications from the several county societies and medical colleges in relation to proposed alterations in the laws relating to the practice of physic and surgery,

RESPECTFULLY REPORT:

That they have had before them the communications from seventeen counties, which they have examined, and find various expressions of opinion on the subject. No communications have been received from any of the medical colleges. The committee would present the following brief statement of the opinions expressed in the communications from the county societies, viz:

1. That it is desirable to vest the power of examining and licensing candidates for the practice of physic and surgery, in persons not engaged in teaching students, or in giving lectures as professors in medical colleges. This is recommended by the county societies of Albany, Delaware, Erie and Ontario.

2. That the county censors should be abolished, and in lieu of them and of the examinations by professors of medical colleges—there should be established boards of censors in each senatorial district, chosen by the State Medical Society.—This is recommended by the county societies of Otsego, Rensselaer, Cortland, Chenango, Steuben, Ontario, Broome, Monroe and Westchester.

3. To abolish the county censors, and to establish a *State Board* of censors to be chosen by the State Medical Society, of one from each Senatorial district, out of persons to be nominated by the respective county societies in each senatorial district. This is recommended by the Monroe County Medical Society.

4. To secure to the county medical societies the power of admitting and expelling members according to their own by-laws, without resorting to the judiciary, and a repeal of all such parts of the general regulations, concerning the practice of physic and surgery as are inconsistent with such power. This is recommended by the County Medical Societies of Rensselaer, Cortland, Otsego, and Monroe.

5. To enlarge the standard of qualifications for medical licensure. This is recommended by the county medical societies of Cortland, Delaware, Steuben and Otsego.

6. To elect a permanent member annually, by the State Medical Society from each senatorial district. This is recommended by the county medical societies of Cortland, Monroe and Rensselaer.

7. In favor of retaining the present organization of the county medical societies, and to ask no alteration in the laws relating to the practice of physic and surgery. This is recommended by the county medical societies of Delaware, Oneida, Herkimer, Steuben, Erie, Albany and Orange.

8. A general acquiescence in the circular of the committee, of which Dr. Davis is chairman, is expressed by the county medical societies of Rensselaer, Broome, (Sullivan,) Otsego, Chenango and Westchester.

The above is a brief summary of the opinions expressed in the various communications of the county societies, although on some of the subjects mentioned, there is some little diversity in the minute details appertaining to each.

No definite action was had by the society upon the subject.

The following preamble and resolution were presented by Dr. Davis and adopted, and

Drs. Davis, McNaughton, and the Secretary, Dr. Van Buren, were appointed a committee to carry out the proposed measure.

*Whereas*, It is believed that a National Convention would be conducive to the elevation of the standard of medical education in the United States, and

*Whereas*, There is no mode of accomplishing so desirable an object, without concert of action on the part of the medical societies, colleges, and institutions of all the States. Therefore,

*Resolved*, That the New York State Medical Society earnestly recommend a national convention of delegates from medical societies and colleges in the whole Union—to convene in the city of New York, on the first Tuesday in May, in the year 1846, for the purpose of adopting some concerted action on the subject set forth in the foregoing preamble.

There can be no doubt that if a National Convention, with full delegations from the various medical societies and colleges could be brought about, that much might be done to regulate medical practice and medical education. Concert of action in all parts of the Union would effect much that is impracticable when the effort is confined to local societies and conventions. We hope that the resolution will be carried into effect.

